# Supplementary material for: Parabrachial nucleus circuit governs neuropathic pain-like behavior
Source: Nat Commun. 2020 Nov 25;11:5974. doi: 10.1038/s41467-020-19767-w (PMC7688648; doi:10.1038/s41467-020-19767-w)
Supplement: Supplementary file 1 — Supplementary Information [file 41467_2020_19767_MOESM1_ESM.pdf]

Parabrachial nucleus circuit governs neuropathic pain-like behavior

Sun et al.

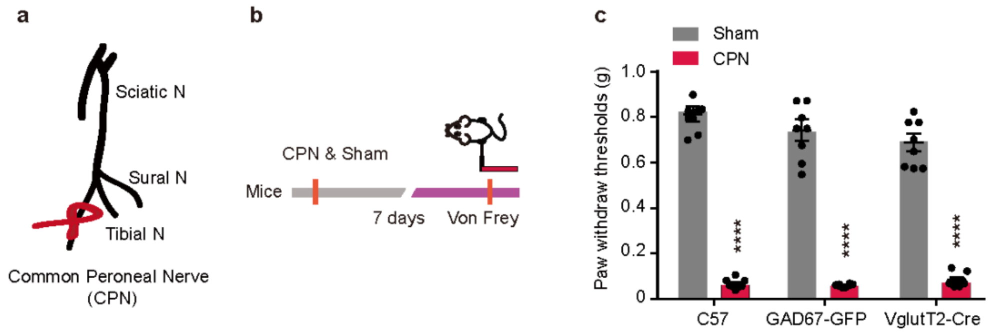

Supplementary Figure 1. Neuropathic pain model induced by ligation of the common peroneal nerve (CPN)

(**a**) Schematic of the CPN model. (**b**) Experimental design and timeline of the behavioral experiment. (**c**) Quantification of the PWT in C57, GAD67-GFP, and VgluT2-ires-Cre mice with or without CPN ligation. Two-way ANOVA followed by Bonferroni's multiple comparisons test: Sham *vs* CPN, C57, ****P <0.0001; GAD67-GFP, ****P <0.0001; VgluT2-Cre, ****P <0.0001; $F_{(2, 42)} = 2.965$; $n = 8$ mice per group. All data are presented as mean ± s.e.m. and error bars represent s.e.m. Source data are provided as a Source Data file.

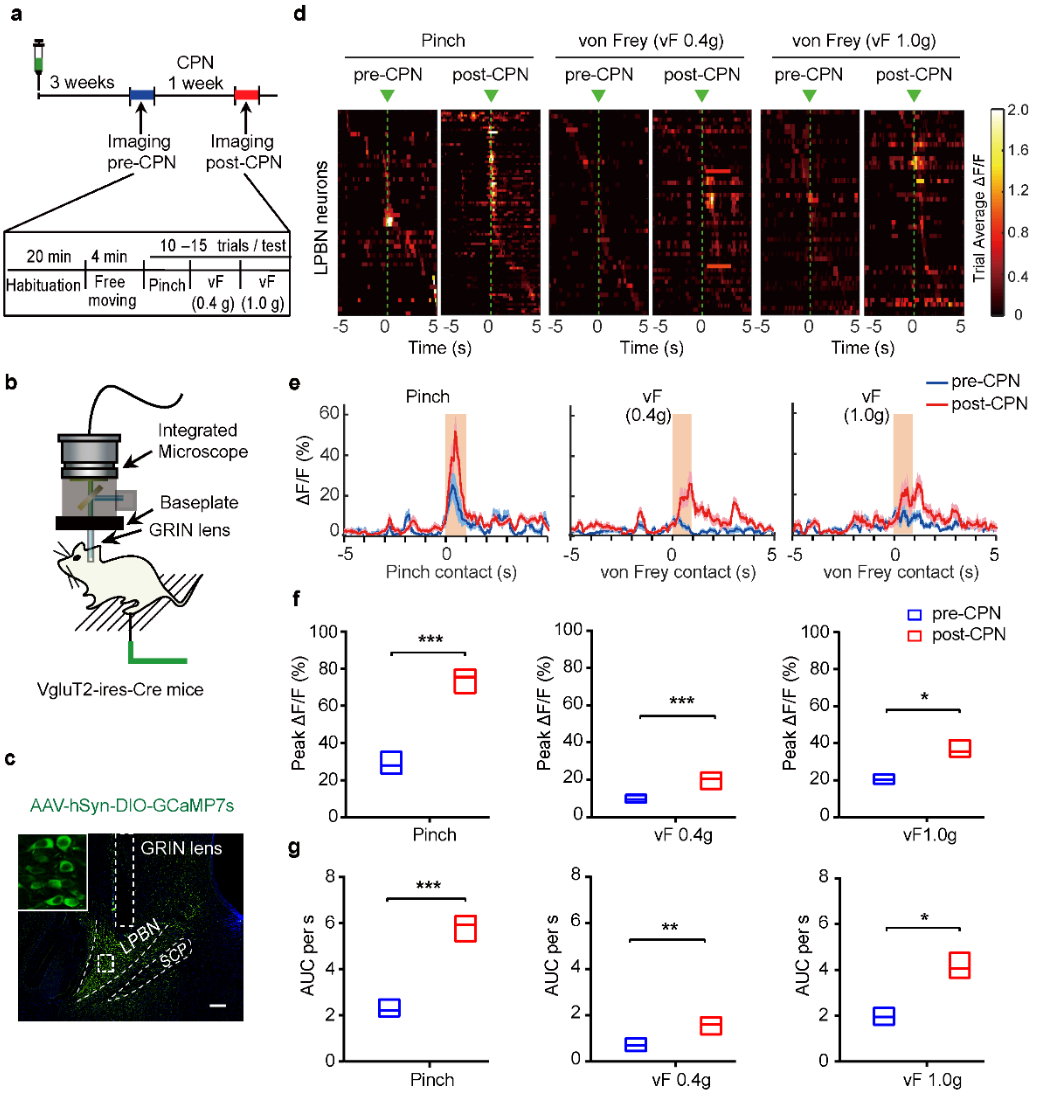

Supplementary Figure 2. Neuropathic pain enhances the activity of glutamatergic LPBN neurons induced by noxious and mechanical stimuli.

(**a**) Experimental timeline for imaging Ca^{2+} activity in VgluT2 LPBN neurons transfected with AAV-DIO-GCaMP7s. (**b**) Diagrams showing Ca^{2+} activity imaging in LPBN VgluT2 neurons using a head-mounted miniaturized microscope. (**c**) Representative image of LPBN neurons transfected with AAV-DIO-GCaMP7s in a Vglut2-ires-Cre mouse. Scale bar, 200 μm. Inset, magnified view of rectangle in image; scale bar, 40 μm. (**d**) Trial-average activity of VgluT2 neurons in response to indicated sensory stimuli before (pre-CPN) and after (post-CPN) CPN ligation. Active Ca^{2+} event traces are aligned to the time when the pinch (n = 56 neurons from 4 mice), von

Frey (0.4 g) (*n* = 56 neurons from 4 mice), or von Frey (1.0 g) (*n* = 47 neurons from 4 mice) stimuli are applied to the hindpaw (green arrowheads). Trial averages (10–15 trials/mouse) are sorted based on peak activity time. Arrowheads indicate onset of stimulation. (**e**) Average response to pinch, von Frey (vF 0.4 g) and von Frey (vF 1.0 g) stimuli before (blue) and after (red) CPN ligation. Number of neurons recorded is the same as in (**d**). (**f, g**) Peak $\Delta F/F$ (**f**) and area under the curve (AUC) per second (**g**) of GCaMP6m signals following pinch, vF 0.4 g and vF 1.0 g stimuli. Wilcoxon Signed Rank Test, (**f**), pinch (pre vs. post), ***P <0.001; *Z value* = -3.506b; *n* = 56 neurons from 4 mice; vF 0.4 g (pre vs. post), ***P <0.001; *Z value* = -4.201b; *n* = 56 neurons from 4 mice; vF 1.0 g (pre vs. post), *P =0.0234; *n* = 47 neurons from 4 mice *Z value* = -2.201b (**g**), pinch (pre vs. post), ***P <0.001; *Z value* = -3.847b; *n* = 56 neurons from 4 mice; vF 0.4 g (pre vs. post), **P =0.002; *Z value* = -3.141b; *n* = 56 neurons from 4 mice; vF 1.0 g (pre vs. post), *P =0.043; *Z value* = -2.027b; *n* = 47 neurons from 4 mice. All data are presented as mean ± s.e.m. and error bars represent s.e.m. Source data are provided as a Source Data file.

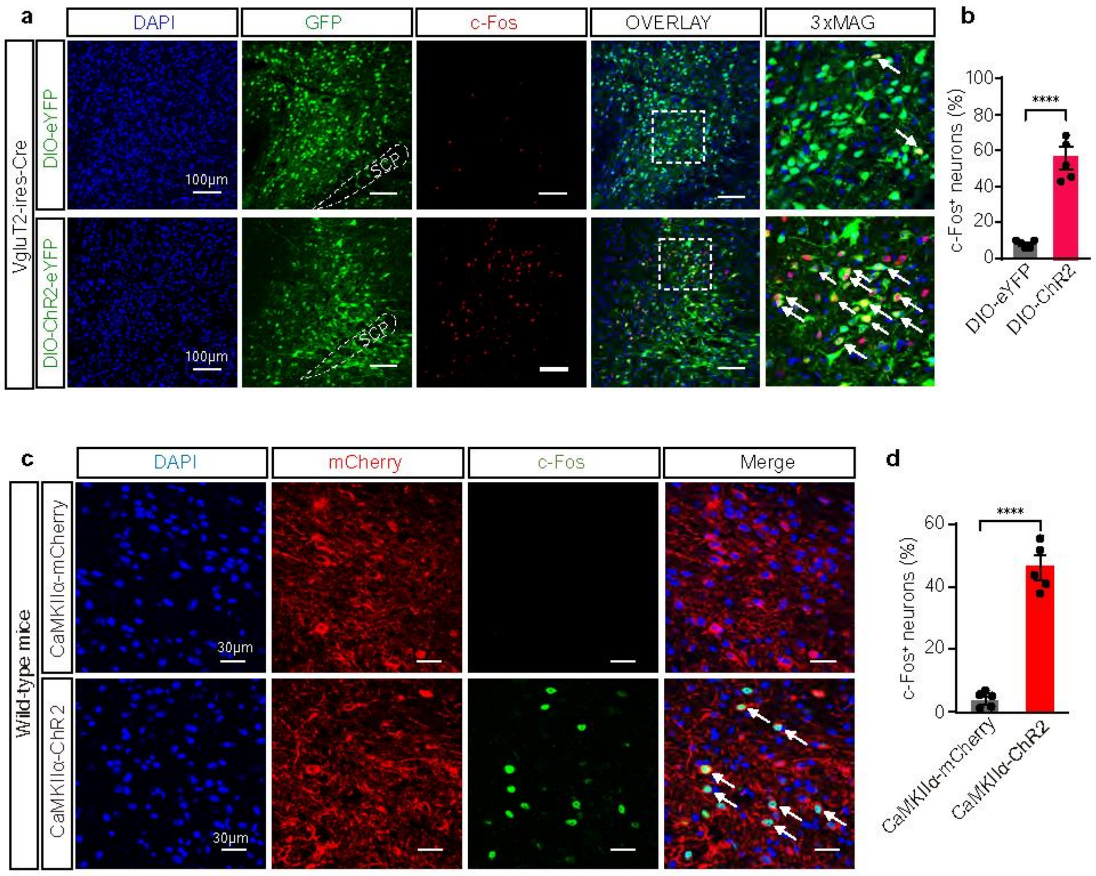

Supplementary Figure 3. Activation and inactivation of glutamatergic LPBN neurons and c-FOS expression levels in the LPBN

(**a**) Representative images showing light stimulation-induced c-Fos expression (red) in the LPBN transfected with DIO- eYFP (upper, green) and DIO-ChR2- eYFP (lower, green) in VgluT2-ires-Cre mice. 3xMAG, magnified view of the boxed areas in the overlay panels. Arrows indicate neurons co-labeled with c-FOS and ChR2- eYFP; blue, DAPI stain; scale bars, 100 μm. (**b**) Proportion of neurons expressing eYFP or ChR2-eYFP that co-label with c-Fos in the LPBN as in **(a)**. Two-tailed unpaired t test, DIO-eYFP *vs* DIO-ChR2, ****P <0.0001, t = 9.161 d.f. = 8. (**c**) Representative images showing light stimulation-induced c-Fos expression (green) in the LPBN transfected with CaMKIIα-mCherry (upper, red) or CaMKIIα-ChR2-mCherry (lower, red) in C57BL/6J mice. Arrows indicate c-Fos expression in CaMKIIα$^+$ neurons; scale bars, 30 μm. (**d**) Proportion of neurons expressing CaMKIIα-mCherry or CaMKIIα-ChR2-mCherry that co-labeled with c-Fos in the LPBN as in **(c)**. Two-tailed unpaired t test,

CaMKIIα-mCherry *vs* CaMKIIα-ChR2, ****P <0.0001, t = 13.73 d.f. = 6. All data are presented as mean ± s.e.m. and error bars represent s.e.m. Source data are provided as a Source Data file.

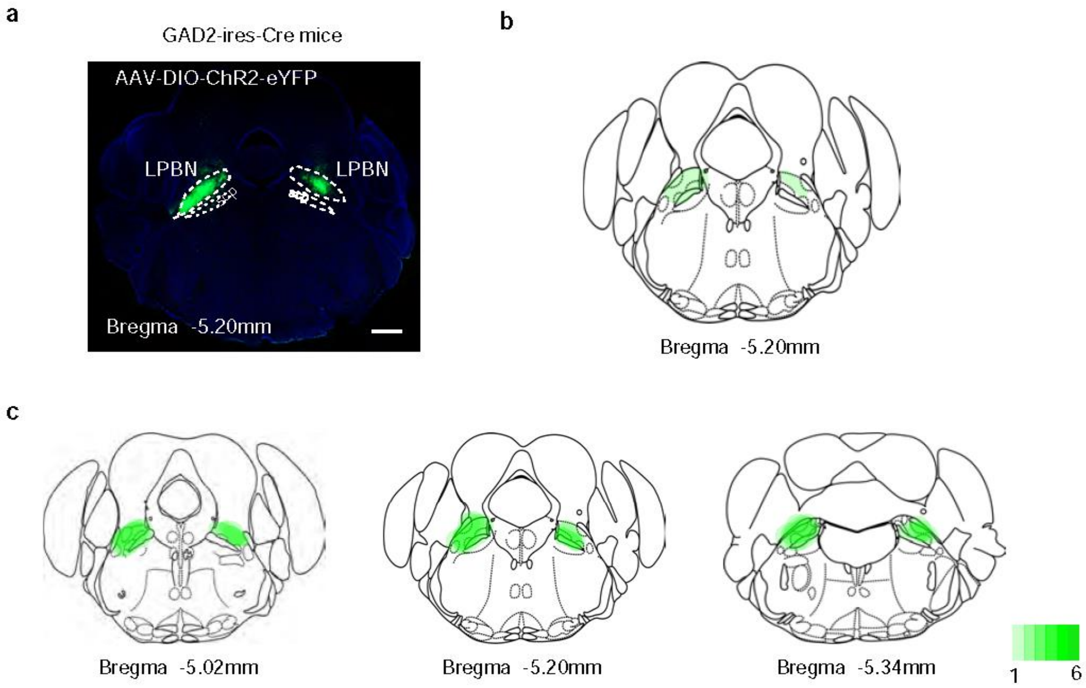

Supplementary Figure 4. Virus infection areas in the LPBN of GAD2-ires-Cre mice.

(**a**) Representative image from a GAD2-ires-Cre mouse bilaterally injected with AAV-DIO-ChR2-eYFP virus in the LPBN. Scale bar, 1 mm. (**b**) Depiction of virus infection area according to the fluorescent image in (**a**). (**c**) Superimposed of virus infection areas at three different coronal levels from six GAD2-ires-Cre mice bilaterally injected with AAV-DIO-ChR2-eYFP virus (1.25 × 10^{12} genomic copies per ml, 80 nl each side) in the LPBN. *n* = 6 mice.

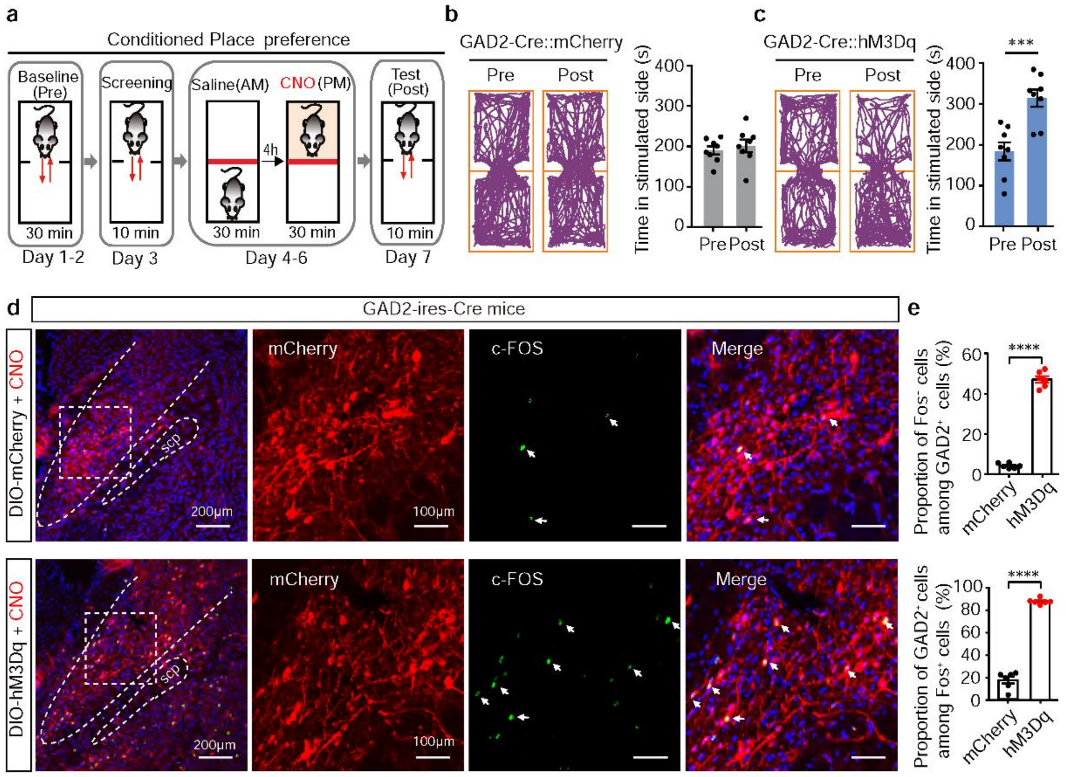

Supplementary Figure 5. Pharmacogenetic activation of GABAergic LPBN neurons induces conditioned place preference (CPP) in CPN-ligated mice.

(a) Schematic of the experimental design for CPP after CPN ligation for 7 days in GAD2-ires-Cre mice. (**b, c**) Examples of tracking maps (left) and quantification of time spent in the preferred chamber (right) in the CPP before (Pre) and after (Post) injection of CNO (5 mg kg^{-1} i.p.) into the LPBN of *GAD2-ires-Cre* mice transfected with DIO-mCherry (b) or DIO-hM3Dq-mCherry (c). Unpaired t test: *P* = 0.5473; *t* = 0.6167; d.f. = 14 (**b**); ****P* = 0.0007; *t* = 4.325; d.f. = 14 (**c**). *n* = 8 mice per group. (**d**) Representative images showing c-Fos expression (green, arrows) and GABAergic neurons transfected with mCherry or hM3Dq-mCherry (red) in the LPBN of GAD2-ires-Cre mice. Scale bar, 200 μm for leftmost panels; 100 μm for other panels; blue, DAPI stain. (**e**) Proportions of c-Fos-positive cells among GAD2-mCherry-expressing neurons (upper) and GAD2-mCherry-positive neurons among c-Fos expressing cells (lower) 1 h after injection of CNO in GAD2-ires-Cre mice. Unpaired t test, (upper) *****P* <0.0001; *t* = 25.17; d.f. = 10; (lower) *****P* <0.0001; *t* = 15.3; d.f. = 10; *n* = 6

mice per group. All data are presented as mean ± s.e.m. and error bars represent s.e.m. Source data are provided as a Source Data file.

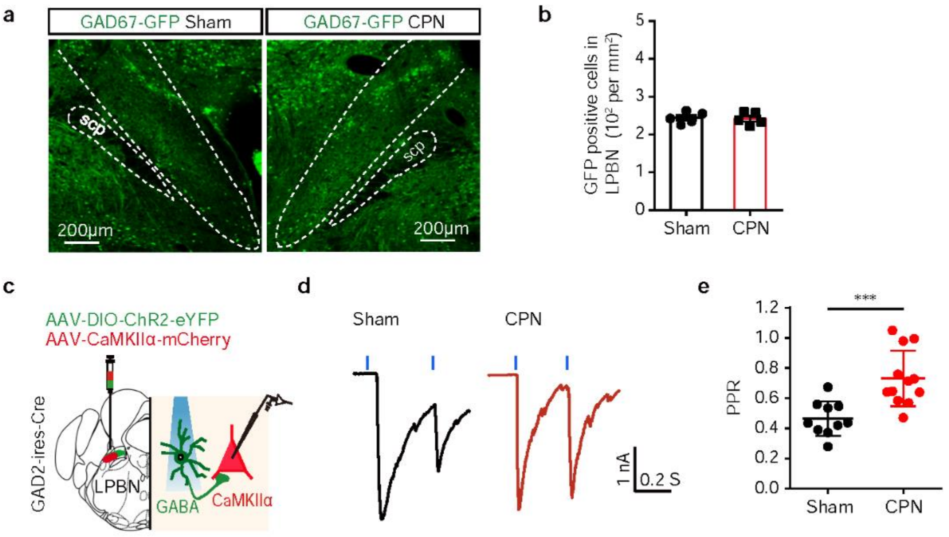

Supplementary Figure 6. Effects of CPN-treatment on GABAergic LPBN neurons.

(a) Representative images showing GAD67-GFP-expressing neurons in the LPBN from sham (left) and CPN-treated (right) GAD67-GFP mice. (**b**) Summarized density of GAD67-GFP-expressing neurons in the LPBN from sham and CPN-treated mice as in (**a**). (**c**) Schematic of patch-clamp recording in brain slices from GAD2-ires-Cre mice transfected with AAV-CaMKIIα-mCherry and AAV-DIO-ChR2-eYFP virus in the LPBN. (**d**) Representative traces of evoked IPSPs in LPBN$^{CaMKII\alpha}$ neurons induced by paired pulses of light stimulation (473 nm, 5-ms pulses at an interval of 200 ms) of LPBN GABAergic neurons in Sham (left) and CPN treated (right) GAD2-ires-Cre mice. (**e**) Summarized paired-pulse ratio (PPR, calculated by dividing the amplitude of the second pulse by that of the first) as in (d). Two-sided unpaired t-test, t = 3.979, d.f. = 20, *** P = 0.0007. n = 10 neurons from 6 mice (Sham) and 12 neurons from 6 mice (CPN). All data are presented as mean ± s.e.m. and error bars represent s.e.m. Source data are provided as a Source Data file.

Supplementary Table 1. Statistical Data

Fig	Comparison	Analysis	P value, F/T/W/U value	N
1c	c-Fos^+ neurons number in the CaMKIIα *vs* GAD67 group with Sham operation or CPN ligation	Two-way ANOVA followed by Bonferroni's multiple comparisons test	Sham *vs* CPN: CaMKIIα, ****P <0.0001; GAD67, P >0.9999; CaMKIIα *vs* GAD67: Sham, P = 0.0647; CPN, ****P<0.0001; F (1, 8) = 364.9;	8 section s from 5 mice
1d	Proportions of CaMKIIα-positive cells and GAD67-GFP-positive cells co-expressed with c-Fos in the LPBN after Sham operation or CPN ligation.	Two-way ANOVA followed by Bonferroni's multiple comparisons test	Sham *vs* CPN: Fos^+/CaMKIIα, ****P <0.0001; Fos^+/Gad67, P = 0.6574; Fos^+/CaMKIIα *vs* Fos^+/Gad67: Sham, P = 0.1660, CPN, ****P <0.0001; F (1, 16) = 93.72	5 section s from 5 mice.
1k	Averaged peak ΔF/F of GCaMP7s and eYFP group with Sham operation or CPN ligation	Two-way ANOVA followed by Bonferroni's multiple comparisons test	Sham *vs* CPN: eYFP, P >0.9999; GCaMP7s, ****P <0.0001; eYFP *vs* GCaMP7s: Sham,*P = 0.0149; CPN, ****P<0.0001; F (1, 8) = 47.62	6 mice per group
1k	Averaged area under the curve (AUC) per second of GCaMP7s and eYFP group with Sham operation or CPN ligation	Two-way ANOVA followed by Bonferroni's multiple comparisons test	Sham *vs* CPN: eYFP, P = 0.8165; GCaMP7s, ****P<0.0001; eYFP *vs* GCaMP7s: Sham, *P = 0.0185; CPN, ****P <0.0001; F (1, 8) = 23.65.	6 mice per group
1n	Averaged peak ΔF/F of GCaMP7s and eYFP group with Sham operation or CPN ligation	Two-way ANOVA followed by Bonferroni's multiple comparisons test	Sham *vs* CPN : eYFP, P >0.9999; GCaMP7s, P >0.9999; eYFP *vs* GCaMP7s: Sham, *P = 0.0164; CPN, P = 0.0623; F (1, 20) = 0.1893.	6 mice per group
1n	Averaged area under the curve (AUC) per second of GCaMP7s and eYFP group with Sham	Two-way ANOVA followed by	Sham *vs* CPN: eYFP, P >0.9999; GCaMP7s, P = 0.5367; eYFP *vs* GCaMP7s:	6 mice per group

	operation or CPN ligation	Bonferroni's multiple comparisons test	Sham, *P = 0.0234;<br>CPN, P = 0.2663;<br>F (1, 20) = 0.7316.	
2d	Paw-withdraw thresholds of ChR2 and eYFP group with Sham operation or CPN ligation	Two-way ANOVA followed by Bonferroni's multiple comparisons test	DIO-eYFP, Sham *vs* CPN:<br>Off, ****P<0.0001;<br>On, ****P <0.0001;<br>DIO-ChR2-eYFP, Sham *vs* CPN:<br>Off, ****P <0.0001;<br>On, P =0.1034;<br>DIO-ChR2-eYFP,Sham:<br>Off *vs* On, ****P <0.0001;<br>DIO-eYFP *vs* DIO-ChR2-eYFP: Sham(On), ****P <0.0001;<br>F (3, 44) = 23.77;	12 mice per group
2e	latency of the thermal paw-withdraw response of ChR2 and eYFP group with Sham operation or CPN ligation	Two-way ANOVA followed by Bonferroni's multiple comparisons test	DIO-eYFP, Off *vs* On,:<br>Sham, P = 0.0614;<br>CPN, P >0.9999;<br>DIO-ChR2-eYFP, Off *vs* On:<br>Sham, ****P <0.0001;<br>CPN,P >0.9999;<br>CPN, DIO-eYFP *vs* DIO-CHR2-eYFP:<br>Off, P >0.9999;<br>On, P >0.9999;<br>F (3, 44) = 109.6.	12 mice per group
2h	Time spent in the stimulated chamber in the RTPA test: pre *vs* light *vs* post	One-way ANOVA followed by Bonferroni's multiple comparisons test	Pre *vs* Light, P >0.9999;<br>Pre *vs* Post, P = 0.4438;<br>Light *vs* Post, P = 0.9724;<br>F (2, 15) = 1.208;	6 mice
2j	Time spent in the stimulated chamber in the RTPA test: pre *vs* light *vs* post	One-way ANOVA followed by Bonferroni's multiple comparisons test	Pre *vs* Light, ****P <0.0001;<br>Pre *vs* Post, ***P = 0.0006;<br>Light *vs* Post, ***P = 0.0002;<br>F (2, 18) = 48.88;	7 mice
3b	PWT in mice injected with AAV-CaMKIIα-ChR2-mCherry and	Two-way ANOVA followed by	CaMKIIα-mCherry *vs* CaMKIIα-ChR2:<br>Pre, P = 0.9523;	10

	AAV-CaMKIIα-mCherry group	Bonferroni's multiple comparisons test	Light, ****P <0.0001;<br>Post, P = 0.8294;<br>CaMKIIα-mCherry:<br>Pre *vs* Light, P = 0.9494;<br>Pre *vs* Post, P = 0.7062;<br>Light *vs* Post, P = 0.5162;<br>CaMKIIα-ChR2:<br>Pre *vs* Light, ****P <0.0001;<br>Light *vs* Post, ****P <0.0001;<br>Pre *vs* Post, P = 0.5665;<br>F (2, 11) = 36.83.	mice per group
3c	Thermal paw-withdrawal latency in mice injected with AAV-CaMKIIα-ChR2-mCherry and AAV-CaMKIIα-mCherry group	Two-way ANOVA followed by Bonferroni's multiple comparisons test	CaMKIIα-mCherry:<br>Pre *vs* Light, P >0.9999;<br>Light *vs* Post, P >0.9999;<br>Pre *vs* Post, P = 0.8261;<br>CaMKIIα-ChR2:<br>Pre *vs* Light, ****P<0.0001;<br>Light *vs* Post, ****P <0.0001;<br>Pre *vs* Post, P = 0.9360;<br>CaMKIIα-mCherry *vs* CaMKIIα-ChR2:<br>Pre, P >0.9999;<br>Light, ****P <0.0001;<br>Post, P = 0.9534;<br>F (2, 21) = 36.83;	10 mice per group
3d	Time spent in the stimulated chamber in the RTPA test: pre *vs* light *vs* post	One-way ANOVA followed by Bonferroni's multiple comparisons test	Pre *vs* Light, P >0.9999;<br>Pre *vs* Post, P = 0.7691;<br>Light *vs* Post, P >0.9999;<br>F (2, 15) = 0.8074;	6 mice
3e	Time spent in the stimulated chamber in the RTPA test: pre *vs* light *vs* post	One-way ANOVA followed by Bonferroni's multiple comparisons test	Pre *vs* Light, ****P <0.0001;<br>Pre *vs* Post, ***P = 0.0006;<br>Light *vs* Post, **P = 0.0013;<br>F (2, 15) = 43.77;	6 mice
3h	Distance moved in the OFT: pre *vs* light *vs* post	Two-way ANOVA followed by	CaMKIIα-mCherry *vs* CaMKIIα-ChR2:<br>Pre, P >0.9999;	5

		Bonferroni's multiple comparisons test	Light, P >0.9999;<br>Post, P = 0.7626);<br>F (2, 12) = 0.6693;	mice per group
3h	Ratio of time spent in the periphery and center in the OFT: pre *vs* light *vs* post	Two-way ANOVA followed by Bonferroni's multiple comparisons test	CaMKIIα-mCherry *vs* CaMKIIα-ChR2<br>Pre, P >0.9999;<br>Light, P >0.9999;<br>Post, P >0.9999;<br>F (2, 12) = 0.3369;	5 mice per group
4b	Paw-withdraw thresholds of eNpHR and mCherry group with Sham operation	Two-way ANOVA followed by Bonferroni's multiple comparisons test	mCherry *vs* eNpHR:<br>Off, P = 0.1361;<br>On, ****P <0.0001;<br>Off *vs* On:<br>mCherry, P = 0.0763;<br>eNpHR (Off *vs* On), *P = 0.0137;<br>F (3, 44) = 49.64;	12 mice per group
4b	Paw-withdraw thresholds of eNpHR and mCherry group with CPN ligation	Two-way ANOVA followed by Bonferroni's multiple comparisons test	mCherry *vs* eNpHR<br>Off, P = 0.2472;<br>On, ****P <0.0001;<br>Off *vs* On:<br>mCherry, P > 0.9999;<br>eNpHR, ****P <0.0001;<br>F (3, 33) = 47.29;	12 mice per group
4c	Latency of the thermal paw-withdraw response of eNpHR and mCherry group with Sham operation	Two-way ANOVA followed by Bonferroni's multiple comparisons test	mCherry *vs* eNpHR:<br>Off, P = 0.6455;<br>On, ****P <0.0001;<br>Off *vs* On:<br>mCherry, P = 0.0602;<br>eNpHR, *P = 0.0107;<br>F (1, 22) = 18.17;	12 mice per group
4c	Latency of the thermal paw-withdraw response of eNpHR and mCherry group with CPN ligation	Two-way ANOVA followed by Bonferroni's multiple comparisons test	mCherry *vs* eNpHR:<br>Off, P = 0.1496;<br>On, ****P <0.0001;<br>Off *vs* On:<br>mCherry, P= 0.8663;<br>eNpHR , ****P <0.0001;<br>F (1, 22) = 96.64;	12 mice per group
4f	Time spent in the stimulated chamber of mCherry group in the	One-way ANOVA followed by Bonferroni's	Pre *vs* Light, P = 0.9418;<br>Pre *vs* Post, P > 0.9999;<br>Light *vs* Post, P> 0.9999;<br>F (2, 12) = 0.6869;	5 mice

	RTPP test: pre *vs* light *vs* post	multiple comparisons test		
4g	Time spent in the stimulated chamber of eNpHR group in the RTPP test: pre *vs* light *vs* post	One-way ANOVA followed by Bonferroni's multiple comparisons test	Pre *vs* Light, **P = 0.0047;<br>Pre *vs* Post, *P = 0.0319;<br>Light *vs* Post, P = 0.9531;<br>F (2, 12) = 8.907;	5 mice
4j	Distance moved in the OFT: pre *vs* light *vs* post	Two-way ANOVA followed by Bonferroni's multiple comparisons test	mCherry *vs* eNpHR:<br>Pre, P = 0.1055;<br>Light, P = 0.4452;<br>Post, P = 0.2445);<br>mCherry:<br>Pre *vs* Light, P > 0.9999;<br>Pre *vs* Post, P = 0.7484;<br>Light *vs* Post, P > 0.9999;<br>eNpHR:<br>Pre *vs* Light, P > 0.9999;<br>Pre *vs* Post, P > 0.9999;<br>Light *vs* Post, P = 0.9286;<br>F (2, 24) = 0.1895.	5 mice per group
4k	Ratio of time spent in the periphery and center in the OFT: pre *vs* light *vs* post	Two-way ANOVA followed by Bonferroni's multiple comparisons test	mCherry *vs* eNpHR:<br>Pre, P > 0.9999;<br>Light, P > 0.9999;<br>Post, P = 0.7001;<br>mCherry:<br>Pre *vs* Light, P = 0.6961;<br>Pre *vs* Post, P > 0.9999;<br>Light *vs* Post, P = 0.9471;<br>eNpHR:<br>Pre *vs* Light, P > 0.9999;<br>Pre *vs* Post, P = 0.7485;<br>Light *vs* Post, P = 0.8849;<br>F (2, 24) = 1.099.	5 mice per group
4l	Distance moved in the OFT: pre *vs* light *vs* post	Two-way ANOVA followed by Bonferroni's multiple comparisons test	mCherry *vs* eNpHR:<br>Pre, P = 0.6075;<br>Light, P > 0.9999;<br>Post, P = 0.8437;<br>mCherry:<br>Pre *vs* Light, P = 0.0520;<br>Pre *vs* Post, P = 0.5881;<br>Light *vs* Post, P = 0.6963;	5 mice per group

			eNpHR:<br>Pre *vs* Light, P > 0.9999;<br>Pre *vs* Post, P = 0.8645;<br>Light *vs* Post, P > 0.9999;<br>F (2, 12) = 0.5950.	
4m	Ratio of time spent in the periphery and center in the OFT: pre *vs* light *vs* post	Two-way ANOVA followed by Bonferroni's multiple comparisons test	mCherry *vs* eNpHR:<br>Pre, P = 0.1582;<br>Light, P > 0.9999;<br>Post, P > 0.9999;<br>F(2, 12) = 0.5402;<br>mCherry:<br>Pre *vs* Light, P = > 0.9999;<br>Pre *vs* Post, P > 0.9999;<br>Light *vs* Post, P > 0.9999;<br>eNpHR:<br>Pre *vs* Light, P > 0.9999;<br>Pre *vs* Post, P = 0.7637;<br>Light *vs* Post, P > 0.9999;<br>F (2, 24) = 0.6976.	5 mice per group
5b	Paw-withdraw thresholds of eNpHR and eYFP group with Sham operation	Two-way ANOVA followed by Bonferroni's multiple comparisons test	eYFP *vs* eNpHR:<br>Off, P >0.9999;<br>On, ****P <0.0001;<br>Off *vs* On:<br>eYFP, P >0.9999;<br>eNpHR, ***P = 0.0005;	12 mice per group
5b	Paw-withdraw thresholds of eNpHR and eYFP group with CPN ligation	Two-way ANOVA followed by Bonferroni's multiple comparisons test	eYFP *vs* eNpHR:<br>Off, P >0.9999;<br>On, ****P <0.0001;<br>Off *vs* On:<br>eYFP, P >0.9999;<br>eNpHR, ****P <0.0001;<br>F (3, 36) = 12.56;	12 mice per group
5c	Latency of the thermal paw-withdraw response of eNpHR and eYFP group with Sham operation	Two-way ANOVA followed by Bonferroni's multiple comparisons test	eYFP *vs* eNpHR:<br>Off, P >0.9999;<br>On, ****P <0.0001;<br>Off *vs* On:<br>eYFP , P = 0.0602;<br>eNpHR, ****P <0.0001;	12 mice per group
5c	Latency of the thermal paw-withdraw response of eNpHR and eYFP group with CPN ligation	Two-way ANOVA followed by	eYFP *vs* eNpHR:<br>Off, P >0.9999;<br>On, ****P <0.0001;<br>Off *vs* On:	12 mice per group

		Bonferroni's multiple comparisons test	eYFP , P = 0.8663;<br>eNpHR, ****P <0.0001;<br>F (3, 66) = 16.42;	
5e	Time spent in the stimulated chamber of eYFP group in the RTPP test: pre *vs* light *vs* post	One-way ANOVA followed by Bonferroni's multiple comparisons test	Pre *vs* Light, P = 0.0672;<br>Pre *vs* Post, P = 0.6642;<br>Light *vs* Post, P = 0.6709;<br>F (2, 15) = 1.754;	6 mice per group
5f	Time spent in the stimulated chamber of eNpHR group in the RTPP test: pre *vs* light *vs* post	One-way ANOVA followed by Bonferroni's multiple comparisons test	Pre *vs* Light, ****P <0.0001;<br>Pre *vs* Post, **P = 0.0032;<br>Light *vs* Post, P = 0.1123;<br>F (2, 12) = 0.09877;	6 mice per group
5h	Distance moved in the OFT: pre *vs* light *vs* post	Two-way ANOVA followed by Bonferroni's multiple comparisons test	eYFP *vs* eNpHR:<br>Pre, P = 0.1215;<br>Light, P = 0.2092;<br>Post, P = 0.8789;<br>eYFP :<br>Pre *vs* Light, P = 0.534;<br>Pre *vs* Post, P = 0.0887;<br>Light *vs* Post, P >0.9999;<br>eNpHR:<br>Pre *vs* Light, P = 0.8560;<br>Pre *vs* Post, P = 0.8108;<br>Light *vs* Post, P >0.9999;<br>F (2, 15) = 0.3595.	6 mice per group
5i	Ratio of time spent in the periphery and center in the OFT: pre *vs* light *vs* post	Two-way ANOVA followed by Bonferroni's multiple comparisons test	eYFP *vs* eNpHR:<br>Pre, P = 0.4586;<br>Light, P = 0.5750;<br>Post, P >0.9999;<br>eYFP:<br>Pre *vs* Light, P >0.9999;<br>Pre *vs* Post, P = 0.5398;<br>Light *vs* Post, P = 0.6181;<br>eNpHR:<br>Pre *vs* Light, P >0.9999;<br>Pre *vs* Post, P = 0.9148;<br>Light *vs* Post, P >0.9999;<br>F (2, 15) = 1.436.	6 mice per group

5j	Distance moved in the OFT: pre *vs* light *vs* post	Two-way ANOVA followed by Bonferroni's multiple comparisons test	eYFP *vs* eNpHR:<br>Pre, P = 0.1549;<br>Light, P = 0.0987;<br>Post, P = 0.2054;<br>eYFP:<br>Pre *vs* Light, P >0.9999;<br>Pre *vs* Post, P = 0.6249;<br>Light *vs* Post, P = 0.7763;<br>eNpHR:<br>Pre *vs* Light, P >0.9999;<br>Pre *vs* Post, P = 0.7828;<br>Light *vs* Post, P >0.9999;<br>F (2, 15) = 0.03084.	6 mice per group
5k	Ratio of time spent in the periphery and center in the OFT: pre *vs* light *vs* post	Two-way ANOVA followed by Bonferroni's multiple comparisons test	eYFP *vs* eNpHR:<br>Pre; Light; Post, P >0.9999;<br>eYFP:<br>Pre *vs* Light, P >0.9999;<br>Pre *vs* Post, P >0.9999;<br>Light *vs* Post, P >0.9999;<br>eNpHR:<br>Pre *vs* Light, P >0.9999;<br>Pre *vs* Post, P >0.9999;<br>Light *vs* Post, P >0.9999;<br>F (2, 15) = 0.1268.	6 mice per group
6b	PWT in vGAT-ChR2-eYFP mice with Sham operation or CPN ligation	Two-way ANOVA followed by Bonferroni's multiple comparisons test	Sham *vs* CPN:<br>Pre, ****P <0.0001;<br>Light, ***P = 0.0003;<br>Post, ****P <0.0001;<br>CPN:<br>Pre *vs* Light, ****P <0.0001;<br>Light *vs* Post, ****P <0.0001;<br>F (1, 8) = 470.5;	8 mice per group
6c	Thermal paw-withdrawal latency in vGAT-ChR2-eYFP mice with Sham operation or CPN ligation	Two-way ANOVA followed by Bonferroni's multiple comparisons test	Sham:<br>Pre *vs* Light, P >0.9999;<br>Pre *vs* Post), P >0.9999;<br>Light *vs* Post, P >0.9999;<br>CPN:<br>Pre *vs* Light, ****P <0.0001;<br>Pre *vs* Post, P >0.9999;<br>Light *vs* Post, ****P <0.0001;	8 mice per group

			Sham *vs* CPN:<br>Pre, ****P <0.0001;<br>Light, P = 0.1903,<br>Post, ****P <0.0001,	
6e	Time spent in the stimulated chamber in the RTPP test: pre *vs* light *vs* post	One-way ANOVA followed by Bonferroni's multiple comparisons test	Pre *vs* Light, P = 0.0572;<br>Pre *vs* Post, P = 0.7494;<br>Light *vs* Post, P = 0.2047;<br>F(2, 15) = 3.3792;	6 mice per group
6g	Time spent in the stimulated chamber in the RTPP test: pre *vs* light *vs* post	One-way ANOVA followed by Bonferroni's multiple comparisons test	Pre *vs* Light, **P = 0.0013;<br>Pre *vs* Post, *P = 0.0153;<br>Light *vs* Post, P = 0.4553;<br>F (2, 15) = 10.49;	7 mice per group
6i	Paw-withdraw thresholds of ChR2 and eYFP group with Sham operation or CPN ligation	Two-way ANOVA followed by Bonferroni's multiple comparisons test	Sham, Off *vs* On:<br>eYFP, P >0.9999;<br>ChR2, P >0.9999;<br>Sham *vs* CPN, Off:<br>eYFP, ****P <0.0001;<br>ChR2, ****P <0.0001;<br>Sham *vs* CPN ,On:<br>eYFP, ****P <0.0001;<br>Off *vs* On, CPN:<br>ChR2, ****P <0.0001;<br>F (3, 12) = 42.14;	10 mice per group
6j	Latency of the thermal paw-withdraw response of ChR2 and eYFP group with Sham operation or CPN ligation	Two-way ANOVA followed by Bonferroni's multiple comparisons test	Sham, Off *vs* On:<br>eYFP, P = 0.9999,<br>ChR2, P = 0.9981;<br>Sham *vs* CPN, Off:<br>eYFP , **P = 0.0022;<br>ChR2, *P = 0.0128;<br>Sham *vs* CPN, On:<br>eYFP, ***P = 0.0004;<br>eYFP *vs* ChR2, On:<br>CPN ,**P = 0.0021;<br>CPN, Off *vs* On:<br>ChR2, **P = 0.0014;<br>F (3, 15) = 5.809;	9 mice per group
6k	Time spent in the stimulated chamber of	One-way ANOVA	eYFP:<br>Pre *vs* Light, P = 0.2122;	6

	ChR2 and eYFP group in the RTPP test: pre *vs* light *vs* post	followed by Bonferroni's multiple comparisons test	Pre *vs* Post, P = 0.5462;<br>Light *vs* Post, P = 0.7658;<br>F (2, 15) = 1.593;<br>ChR2:<br>Pre *vs* Light, **P = 0.0068;<br>Pre *vs* Post, *P = 0.0485;<br>Light *vs* Post, ns, P = 0.5901;<br>F (2, 15) = 6.951;	mice per group
6l	Distance moved in the OFT: pre *vs* light *vs* post	Two-way ANOVA followed by Bonferroni's multiple comparisons test	eYFP *vs* ChR2:<br>Pre, P = 0.2524;<br>Light, P = 0.1670;<br>Post, P = 0.083;<br>eYFP:<br>Pre *vs* Light, P > 0.9999;<br>Pre *vs* Post, P =0.4933;<br>Light *vs* Post, P > 0.9999;<br>ChR2:<br>Pre *vs* Light, P > 0.9999;<br>Pre *vs* Post, P > 0.9999;<br>Light *vs* Post, P > 0.9999;<br>F (2, 12) = 0.05689.	5 mice per group
6l	Ratio of time spent in the periphery and center in the OFT: pre *vs* light *vs* post	Two-way ANOVA followed by Bonferroni's multiple comparisons test	eYFP *vs* ChR2:<br>Pre; Light; Post, P > 0.9999;<br>eYFP:<br>Pre *vs* Light, P > 0.9999;<br>Pre *vs* Post, P > 0.9999;<br>Light *vs* Post, P > 0.9999;<br>ChR2:<br>Pre *vs* Light, P > 0.9999;<br>Pre *vs* Post, P > 0.9999;<br>Light *vs* Post, P > 0.9999;<br>F (2, 12) = 0.07768;	5 mice per group
6n	Time-course of the CPN ligation in PWT and the effect of pharmacogenetic activation of GABAergic LPBN neurons	Two-way ANOVA followed by Bonferroni's multiple comparisons test	mCherry *vs* hM3Dq CNO:<br>Baseline, P = 0.9862;<br>day 3, P = 0.9994;<br>day 7, P = 0.9989;<br>day 8, P = 0.0886;<br>day 9, *P = 0.0210;<br>day 10, ****P <0.0001;<br>day 11, ****P <0.0001;<br>mCherry *vs* hM3Dq CNO + PTX:	8 mice per group

			Baseline, P = 0.7085;<br>day 3, P = 0.9872;<br>day 7, P >0.9999;<br>day 8, P = 0.2474;<br>day 9, *P = 0.0212;<br>day 10, ****P <0.0001;<br>day 11 (PTX), P = 0.9512.<br>F (6, 49) = 5.333;	
7b	Paw-withdraw thresholds of GtACR1 and eYFP group	Two-way ANOVA followed by Bonferroni's multiple comparisons test	eYFP *vs* GtACR1:<br>Off, P = 0.6941;<br>On, ****P <0.0001;<br>Off *vs* On:<br>eYFP, P >0.9999;<br>GtACR1, ****P <0.0001;<br>F(1, 18) = 22.26;	10 mice per group
7c	Latency of the thermal paw-withdraw response of GtACR1 and eYFP group	Two-way ANOVA followed by Bonferroni's multiple comparisons test	eYFP *vs* GtACR1:<br>Off, P >0.9999;<br>On, **P = 0.0075;<br>Off *vs* On:<br>eYFP, P >0.9999;<br>GtACR1, ***P = 0.001;<br>F(1, 18) = 7.521;	10 mice per group
7e	Time spent in the stimulated chamber of GtACR1 and eYFP group in the RTPA test: pre *vs* light *vs* post	One-way ANOVA followed by Bonferroni's multiple comparisons test	eYFP:<br>Pre *vs* Light, P = 0.9995;<br>Pre *vs* Post, P = 0.7081;<br>Light *vs* Post, P = 0.6900;<br>F (2, 15) = 0.4445;<br>GtACR1:<br>Pre *vs* Light, ****P <0.0001;<br>Pre *vs* Post, *P = 0.0178;<br>Light *vs* Post, *P = 0.0405;<br>F (2, 18) = 16.35;	eYFP: n = 6; GtACR1: n = 7
7f	Distance moved in the OFT: pre *vs* light *vs* post	Two-way ANOVA followed by Bonferroni's multiple comparisons test	eYFP *vs* GtACR1:<br>Pre, P = 0.0610;<br>Light, P = 0.3569;<br>Post, P = 0.1740;<br>eYFP:<br>Pre *vs* Light, P > 0.9999;<br>Pre *vs* Post, P = 0.7484;<br>Light *vs* Post, P > 0.9999;<br>GtACR1:<br>Pre *vs* Light, P > 0.9999;	5 mice per group

			Pre *vs* Post, P > 0.9999;<br>Light *vs* Post, P = 0.9286;<br>F (2, 24) = 0.1895;	
7f	Ratio of time spent in the periphery and center in the OFT: pre *vs* light *vs* post	Two-way ANOVA followed by Bonferroni's multiple comparisons test	eYFP *vs* GtACR1:<br>Pre, P = 0.7191;<br>Light, P > 0.9999;<br>Post, P = 0.7700;<br>eYFP:<br>Pre *vs* Light, P > 0.9999;<br>Pre *vs* Post, P = 0.6744;<br>Light *vs* Post, P > 0.9999;<br>GtACR1:<br>Pre *vs* Light, P > 0.9999;<br>Pre *vs* Post, P = 0.8193;<br>Light *vs* Post, P = 0.9571;<br>F (2, 24) = 1.619;	5 mice per group
7h	c-Fos^+ neurons number in the GtACR1 *vs* eYFP group	unpaired t test	****P <0.0001;<br>t = 20.6;<br>d.f. = 12;	6 mice per group
7i	c-Fos^+ neurons number in the GtACR1 *vs* eYFP group	unpaired t test	P = 0.7076;<br>t = 0.386;<br>d.f. = 10;	6 mice per group
7j	Proportion of c-Fos-positive cells that co-express GAD2-eYFP or CaMKIIα$^+$ cells	unpaired t test	****P <0.0001;<br>t = 24.98;<br>d.f. = 10;	6 mice per group
8j	Time course of summarized changes in spike frequency (Hz) of LPBN CaMKIIα neurons induced by light stimulation of GABAergic LPBN neurons	One-way ANOVA followed by Bonferroni's multiple comparisons test	Average of –5 to –1 s *vs* 1 s, ***P = 0.0001;<br>average of –5 to –1 s *vs* 2, 3, 4, 5, 6, 7, 8, and 9 s, ****P <0.0001;<br>average of –5 to –1 s *vs* 10 s, *P = 0.0462;<br>average of –5 to –1 s *vs* 11, 12, 13, 14, and 15 s,<br>P >0.9999;<br>F(15, 144) = 37.57	10 neurons from 3 mice
8k	Averaged firing frequency of LPBN CaMKIIα neurons	One-way ANOVA followed by	Before *vs* During, ****P <0.0001; Before *vs* After 0–5s, ****P <0.0001; Before	10 neuron

		Bonferroni's multiple comparisons test	*vs* After 5–10s, P >0.9999; During *vs* After 0–5s, P >0.9999; During *vs* After 5–10s, ****P <0.0001; After 0–5s *vs* After 5–10s, ****P <0.0001; F (3, 196) = 128.6.	s from 3 mice
9b	Time-course of PWT changes	Two-way ANOVA followed by Bonferroni's multiple comparisons test	hM3Dq-Saline *vs* hM3Dq-CNO: Baseline, 0, 1, P >0.9999; 3, *P = 0.0153; 5, 7, 9, 11, 13, ****P <0.0001; 21, **** P <0.0001; 30, ****P <0.0001; F (1, 8) = 138.4;	5 mice per group
9e	Time spent in the stimulated chamber in the CPA test	unpaired t test	mCherry: P = 0.4865; t = 0.7296. hM3Dq: ***P = 0.0004; t = 5.277.	8 mice per group
9g	Time-course of PWT changes	Two-way ANOVA followed by Bonferroni's multiple comparisons test	DIO-eYFP + CaMKIIα-mCherry CNO *vs* DIO-ChR2 + CaMKIIα-hM3Dq CNO : Baseline, P = 0.9005; 1, P = 0.9954, 3, *P = 0.0328; 5, 7, 9, 11, 13, 15, 17, ****P <0.0001; day 21 On, P = 0.3468; day 21 Off, ****P <0.0001;	8 mice er group
9i	Time-course of PWT changes	Two-way ANOVA followed by Bonferroni's multiple comparisons test	mCherry-CPN *vs* hM3Dq-CPN: Baseline, P > 0.9999; 1, P > 0.9999; 3, **P = 0.0063; 5, **P = 0.0013; 7, 9, 11, 13, 15, 28, ****P <0.0001; hM3Dq-Sham *vs* hM3Dq-CPN): Baseline, P > 0.9999; 1, 3,5, 7, 9, 11, 13, 15, 28, P > 0.9999;	5 mice per group

9k	c-Fos-positive cells in the LPBN from different groups	One-way ANOVA followed by Bonferroni's multiple comparisons test	mCherry Sham *vs* mCherry CPN, ****P <0.0001; mCherry Sham *vs* hM3Dq Sham, P = 0.9235; mCherry Sham *vs* hM3Dq CPN, P = 0.4521; mCherry CPN *vs* hM3Dq Sham, ****P <0.0001; mCherry CPN *vs* hM3Dq CPN, ****P < 0.0001; hM3Dq Sham *vs* hM3Dq CPN, P > 0.9999. F (3, 12) = 42.66.	4 mice per group

Supplementary Table 2. Key Resources

REAGENT or RESOURCE	SOURCE	IDENTIFIER
**Primary Antibodies**		
Guinea pig anti-c-Fos	SYSY	Cat# 226004
Rabbit anti-c-Fos	SYSY	Cat# 226003
Rabbit anti-CaMKIIα	Abcam	Cat# ab52476
Mouse anti-Vgat	SYSY	Cat# 131011
DsRed living colors	Takara	Cat# 632496
**Secondary Antibodies**		
Donkey anti-Guinea pig Alexa Fluor 488	Jackson ImmunoResearch Laboratories, Inc.	Cat# 706-545-148
Donkey anti-rabbit Cy3	Jackson ImmunoResearch Laboratories, Inc.	Cat# 711-165-152
Donkey anti-Guinea pig Cy3	Jackson ImmunoResearch Laboratories, Inc.	Cat# 706-165-148
Donkey anti-rabbit Alexa Fluor 488	Jackson ImmunoResearch Laboratories, Inc.	Cat# 711-545-152
Donkey anti-mouse Alexa Fluor 488	Invitrogen	Cat# A21202
**RNAscope in situ hybridization**		
RNAscope Multiplex Fluorescent Reagent Kit v2	Advanced Cell Diagnostics	Cat# 323100

RNAscope Probe-Mm-Gad1-C2	Advanced Cell Diagnostics	Cat# 400951
RNAscope Probe-Mm-Pvalb-C3	Advanced Cell Diagnostics	Cat# 421931
RNAscope Probe-Mm-Slc17a6-C3	Advanced Cell Diagnostics	Cat# 319171
RNAscope Probe-Mm-Cck-C1	Advanced Cell Diagnostics	Cat# 402271
RNAscope Probe-Mm-Sst-C1	Advanced Cell Diagnostics	Cat# 404631
RNAscope Negative control probe dapB	Advanced Cell Diagnostics	Cat# 310043
RNAscope Positive control probe Mm-Ppib	Advanced Cell Diagnostics	Cat# 313911
**Virus Strains**		
AAV2/9-CamKIIα-hChR2(H134R)-mCherry	Shanghai SunBio Biomedical technology Co.	N/A
AAV2/9-CamKIIα-mCherry	Shanghai SunBio Biomedical technology Co.	N/A
AAV2/8-EF1α-DIO-eYFP	Shanghai Taitool Bioscience Co.	Cat# S0196-8
AAV2/9-EF1α-DIO-ChR2-eYFP	Shanghai Taitool Bioscience Co.	Cat# S0199

AAV2/9-CamKIIα-eNpHR3.0-mCherry	Shanghai SunBio Biomedical technology Co.	Cat# S0464-9
AAV2/9-EF1α-DIO-eNpHR3.0-eYFP	Shanghai Taitool Bioscience Co.	Cat# S0178-9
AAV2/9-hSyn-DIO-hM3Dq-mCherry	Shanghai SunBio Biomedical technology Co.	Cat# S0144-9-H20
AAV2/9-CaMKIIα-hM3Dq-mCherry	Shanghai Taitool Bioscience Co.	Cat# S0484-9
AAV2/8-EF1α-DIO-mCherry	Shanghai SunBio Biomedical technology Co	N/A
AAV9-EF1α-DIO-hGtACR1-P2A-eYFP-WPRE	Shanghai Taitool Bioscience Co.	Cat# S0311-8
AAV-CAG-DIO-TVA-eGFP	BrainVTA Wuhan	Cat# AAV-903
AAV-CAG-DIO-RG	BrainVTA Wuhan	Cat# AAV-902
RV-EnvA-DsRed	BrainVTA Wuhan	Cat# RV-306
AAV2/9-hSyn-DIO-GCaMP7s-WPRE	Shanghai Taitool Bioscience Co.	Cat# S0590-9-H20
**Chemicals**		
Clozapine N-oxide	Enzo Life Sciences, Inc.	Cat # NS105-0025
DAPI	Sigma-Aldrich	N/A

**Experimental Animals**		
vGAT-ChR2(H134R)-eYFP	The Jackson Laboratory	JAX014548
GAD2-ires-Cre (B6N.Cg-Gad2$^{tm2(cre)Zjh}$/J)	The Jackson Laboratory	JAX019022
C57BL6/J	Shanghai SLAC Laboratory Animal Co. Ltd	nttp://www.slaccas.com/
VgluT2-ires-Cre (Slc17a6$^{tm2(cre)Lowl}$/J)	The Jackson Laboratory	JAX016963
Gad67-GFP	From the *Takeshi Kaneko* laboratory of Kyoto University	N/A
**Software and Code**		
ANY-Maze software 5.3	Global Biotech Inc.	nttp://www.anymaze.co.uk/
Plantar Test apparatus	IITC Life Science Inc.	nttp://www.iitcinc.com/
Implantable Optical Fibers	Anlai, Ningbo	nttp://www.anilab.cn/
Image J	NIH	nttps://imagej.nih.gov/ij/index.html;

GraphPad Prism 6	GraphPad Software	https://www.graphpad.com/scientificsoftware/prism/;RRID: SCR_002798
Fiber photometry system	Thinker Tech Nanjing Bioscience Inc.	N/A
MatLab R2016a	MathWorks	tps://www.mathworks.com/products.html; RRID:SCR_001622
