# Supplementary material for: Parabrachial nucleus circuit governs neuropathic pain-like behavior
Source: Nat Commun. 2020 Nov 25;11:5974. doi: 10.1038/s41467-020-19767-w (PMC7688648; doi:10.1038/s41467-020-19767-w)
Supplement: Supplementary file 3 — Reporting Summary [file 41467_2020_19767_MOESM3_ESM.pdf]

nature research

Corresponding author(s): Li Sun, Shumin Duan

Last updated by author(s): Oct 14, 2020

Reporting Summary

Nature Research wishes to improve the reproducibility of the work that we publish. This form provides structure for consistency and transparency in reporting. For further information on Nature Research policies, see our Editorial Policies and the Editorial Policy Checklist.

Statistics

For all statistical analyses, confirm that the following items are present in the figure legend, table legend, main text, or Methods section.

n/a	Confirmed	
☐	☒	The exact sample size (n) for each experimental group/condition, given as a discrete number and unit of measurement
☐	☒	A statement on whether measurements were taken from distinct samples or whether the same sample was measured repeatedly
☐	☒	The statistical test(s) used AND whether they are one- or two-sided<br>*Only common tests should be described solely by name; describe more complex techniques in the Methods section.*
☐	☒	A description of all covariates tested
☐	☒	A description of any assumptions or corrections, such as tests of normality and adjustment for multiple comparisons
☐	☒	A full description of the statistical parameters including central tendency (e.g. means) or other basic estimates (e.g. regression coefficient) AND variation (e.g. standard deviation) or associated estimates of uncertainty (e.g. confidence intervals)
☐	☒	For null hypothesis testing, the test statistic (e.g. F, t, r) with confidence intervals, effect sizes, degrees of freedom and P value noted<br>*Give P values as exact values whenever suitable.*
☒	☐	For Bayesian analysis, information on the choice of priors and Markov chain Monte Carlo settings
☒	☐	For hierarchical and complex designs, identification of the appropriate level for tests and full reporting of outcomes
☐	☒	Estimates of effect sizes (e.g. Cohen's d, Pearson's r), indicating how they were calculated

Our web collection on statistics for biologists contains articles on many of the points above.

Software and code

Policy information about availability of computer code

Data collection	Confocal imaging (Olympus, System FV1200; VS120), pClamp 10.4 (RRID: SCR_011323) for whole-cell patch-clamp recordings; ANY-maze software 5.3 via a webcam (Logitech web-camera) for video-recording the open field test, place preference and avoidance behaviors. Fiber photometry system (Thinker Tech Nanjing Bioscience Inc.) Field Mate Laser power meter (Coherent Inc.) Plantar Test apparatus (IITC Life Science Inc.) for Hargreaves test. ProView implant lens probe (~6.1mm length, 0.5mm diameter; Inscopix, #1050-002211); baseplate of a miniaturized integrated fluorescent microscope (Inscopix, #1050-002192) was fixed on top of the microendoscopein well-labeling animals.
Data analysis	Data were graphed and analyzed using Graphpad software Prism 6 (RRID: SCR_002798), Image J 1.48V (RRID: SCR_003070),FIJI (image J-win64), Olympus FV10-ASW 4.0a Viewer, Adobe Photoshop CC 2017, Adobe Illustrator CS6 (version 16), Microsoft office 2013, Adobe Acrobat Pro 2017, IBM SPSS_26.0. MATLAB R2016a (RRID:SCR_001622) was used to fiber photometry and mouse movements for place preference and avoidance tests. Igor pro software was used to analyze electrophysiological data. SPSS_26.0 software was used to analyze Levene' s test of Equality of Error Variances to examine whether our data are suitable for using parametric statistics. Calcium imaging videos were analyzed and neuronal signals were detected using Imaging Data Processing (Inscopix) and custom-written scripts in Matlab following published algorithms (PCA-ICA).

For manuscripts utilizing custom algorithms or software that are central to the research but not yet described in published literature, software must be made available to editors and reviewers. We strongly encourage code deposition in a community repository (e.g. GitHub). See the Nature Research guidelines for submitting code & software for further information.

Data

Policy information about availability of data

All manuscripts must include a data availability statement. This statement should provide the following information, where applicable:

- Accession codes, unique identifiers, or web links for publicly available datasets
- A list of figures that have associated raw data
- A description of any restrictions on data availability

Source data are provided with this paper. All data are contained in the main text and the supplementary materials, and are available from the corresponding author upon reasonable request. The source data underlying Figs. 1c, d, f, h, k, n; 2d, e, h, j; 3b – e, 3h; 4b, c, f, g , j – m; 5b, c, e, f, h – k; 6b, c, e, g, i – l, n; 7b, c, f, h, i, j; 8f, g, j, k; 9b, e, g, i ,k and Supplementary Figs. 1c; 2f, g; 3b, d; 5b, c, e and 6b, e are provided as a Source Data file. Allen Brain Atlas (http://www.brain-map.org).

Field-specific reporting

Please select the one below that is the best fit for your research. If you are not sure, read the appropriate sections before making your selection.

☒ Life sciences ☐ Behavioural & social sciences ☐ Ecological, evolutionary & environmental sciences

For a reference copy of the document with all sections, see nature.com/documents/nr-reporting-summary-flat.pdf

Life sciences study design

All studies must disclose on these points even when the disclosure is negative.

Sample size	The sample sizes were determined by common practice in the field. The statistical significance of the results we obtained justifies the sample size we used. PMID: 26727549, PMID: 22895716, PMID: 24613308, PMID: 20673755
Data exclusions	Animals in which histological examination showed that viral targeting or the position of implanted / cannulas were in the incorrect location were excluded from analysis.
Replication	The experiment for each experiment was successfully repeated for at least two times.
Randomization	Mice were randomly assigned to light or no light stimulation, drug delivery, and CPN or Sham for in vivo experiments.
Blinding	Blinding between CPN and sham conditions was conducted for a subset of behavioral experiments involving optogenetic and studies of the LPBN. Data analysis were performed in a manner blinded to treatment assignments in all behavioral experiments.

Reporting for specific materials, systems and methods

We require information from authors about some types of materials, experimental systems and methods used in many studies. Here, indicate whether each material, system or method listed is relevant to your study. If you are not sure if a list item applies to your research, read the appropriate section before selecting a response.

Materials & experimental systems

n/a	Involved in the study	
☐	☒	Antibodies
☒	☐	Eukaryotic cell lines
☒	☐	Palaeontology and archaeology
☐	☒	Animals and other organisms
☒	☐	Human research participants
☒	☐	Clinical data
☒	☐	Dual use research of concern

Methods

n/a	Involved in the study	
☒	☐	ChIP-seq
☒	☐	Flow cytometry
☒	☐	MRI-based neuroimaging

Antibodies

Antibodies used	Primary Antibodies: anti-c-Fos (1:500, Guinea pig, SYSY, Cat# 226004), anti-c-fos antibody (rabbit, 1:1000, SYSY, Cat#: 226003, anti-CaMKIIα (1:1000, rabbit, Abcam, Cat# ab52476), anti-vGAT (1:800, mouse, SYSY, Cat# 131011).<br>Secondary Antibodies: Donkey anti-Guinea pig Alexa Fluor 488 (1:1000, Jackson, Cat# 706-545-148); Donkey anti-rabbit Cy3 (1:1000, Jackson, Cat# 711-165-152); Donkey anti-Guinea pig Cy3 (1:1000, Jackson, Cat# 706-165-148); Donkey anti- rabbit Alexa Fluor 488 (1:1000, Jackson, Cat# 711-545-152); Donkey anti-mouse Alexa Fluor 488(1:1000, Invitrogen, Cat# A21202).
Validation	For anti-c-Fos, Guinea pig, https://www.sysy.com/products/c-fos/facts-226004.php; website states that the antibody has been validated in WB, ICC, IHC, IHC-P, FAC and reacts with human, rat, mouse, monkey, ape, cow, dog, pig; validation on publication:

PMID: 30801002.
For anti-c-Fos, rabbit, https://www.sysy.com/products/c-fos/facts-226003.php; website states that the antibody has been validated in WB, ICC, IHC, IHC-P and tested in human, rat, mouse, monkey, ape, cow, dog, pig; validation on publication: PMID: 331257028.
For anti-CaMKIIα, rabbit, https://www.abcam.com/camkii-alpha-antibody-ab87597.html; suitable for ICC/IF, WB, IHC and reacts with mouse, rat, human; validation on publication: PMID: 31202540.
For anti-vGAT, mouse, https://www.sysy.com/products/vgat/facts-131011.php; website states that the antibody has been validated in WB, IP, ICC, IHC, IHC-P, FACS, EM and reacts with: human, rat, mouse, Guinea pig, monkey; validation on publication: PMID:31324538.
Donkey anti-Guinea pig Alexa Fluor 488, website states that the antibody has been tested for IHC, PMID: 30375975, RRID: AB_2340472.
Donkey anti-rabbit Cy3, website states that the antibody has been tested for IHC. PMID: 31719165, RRID: AB_2307443.
Donkey anti-Guinea pig Cy3, website states that the antibody has been tested for IHC. PMID: 30318409, RRID: AB_2340460.
Donkey anti-rabbit Alexa Fluor 488, website states that the antibody has been tested for IHC. PMID: 31801252, RRID: AB_2313584.
Donkey anti-mouse Alexa Fluor 488, website states that the antibody has been tested for IHC. PMID: 28266911, PMID: 27214567.

Animals and other organisms

Policy information about studies involving animals; ARRIVE guidelines recommended for reporting animal research

Laboratory animals	Adult male (8–10 weeks, 20–35 g) VgluT2-ires-Cre (Slc17a6tm2(cre)Lowl/J) (JAX016963), vGAT-ChR2 (H134R)-eYFP (JAX014548), GAD2-ires-Cre (B6N.Cg-Gad2tm2(cre)Zjh/J) (JAX019022), Gad67-GFP (from the Takeshi Kaneko laboratory of Kyoto University), and C57BL/6J (wild-type, from the Shanghai SLAC Laboratory Animal Co. Ltd) mice were used.
Wild animals	No wild animals were used.
Field-collected samples	No sample was collected from the field.
Ethics oversight	All experiments were conducted in accordance with the guidelines for the care and use of laboratory animals of Zhejiang University and were approved by the Animal Advisory Committee at Zhejiang University (ZJU).

Note that full information on the approval of the study protocol must also be provided in the manuscript.
